# Maternal intake of high n-6 polyunsaturated fatty acid diet during pregnancy causes transgenerational increase in mammary cancer risk in mice

**DOI:** 10.1186/s13058-017-0866-x

**Published:** 2017-07-03

**Authors:** Nguyen M. Nguyen, Fabia de Oliveira Andrade, Lu Jin, Xiyuan Zhang, Madisa Macon, M. Idalia Cruz, Carlos Benitez, Bryan Wehrenberg, Chao Yin, Xiao Wang, Jianhua Xuan, Sonia de Assis, Leena Hilakivi-Clarke

**Affiliations:** 1 Department of Oncology, Georgetown University, Research Building, Room E407, 3970 Reservoir Road, NW, Washington, DC 20057 USA; 2 UTHealth Graduate School of Biomedical Sciences, The University of Texas MD Anderson Cancer Center, Houston, TX USA; 3 Department of Electrical and Computer Engineering, Virginia Tech, Arlington, VA USA

**Keywords:** Breast cancer, Transgenerational, n-6 Polyunsaturated fatty acids, Primordial germ cells, Maternal diet

## Abstract

**Background:**

Maternal and paternal high-fat (HF) diet intake before and/or during pregnancy increases mammary cancer risk in several preclinical models. We studied if maternal consumption of a HF diet that began at a time when the fetal primordial germ cells travel to the genital ridge and start differentiating into germ cells would result in a transgenerational inheritance of increased mammary cancer risk.

**Methods:**

Pregnant C57BL/6NTac mouse dams were fed either a control AIN93G or isocaloric HF diet composed of corn oil high in n-6 polyunsaturated fatty acids between gestational days 10 and 20. Offspring in subsequent F1–F3 generations were fed only the control diet.

**Results:**

Mammary tumor incidence induced by 7,12-dimethylbenz[a]anthracene was significantly higher in F1 (*p* < 0.016) and F3 generation offspring of HF diet-fed dams (*p* < 0.040) than in the control offspring. Further, tumor latency was significantly shorter (*p* < 0.028) and burden higher (*p* < 0.027) in F1 generation HF offspring, and similar trends were seen in F3 generation HF offspring. RNA sequencing was done on normal mammary glands to identify signaling differences that may predispose to increased breast cancer risk by maternal HF intake. Analysis revealed 1587 and 4423 differentially expressed genes between HF and control offspring in F1 and F3 generations, respectively, of which 48 genes were similarly altered in both generations. Quantitative real-time polymerase chain reaction analysis validated 13 chosen up- and downregulated genes in F3 HF offspring, but only downregulated genes in F1 HF offspring. Ingenuity Pathway Analysis identified upregulation of Notch signaling as a key alteration in HF offspring. Further, knowledge-fused differential dependency network analysis identified ten node genes that in the HF offspring were uniquely connected to genes linked to increased cancer risk (*ANKEF1*, *IGFBP6*, *SEMA5B*), increased resistance to cancer treatments (*SLC26A3*), poor prognosis (*ID4*, *JAM3*, *TBX2*), and impaired anticancer immunity (*EGR3*, *ZBP1*).

**Conclusions:**

We conclude that maternal HF diet intake during pregnancy induces a transgenerational increase in offspring mammary cancer risk in mice. The mechanisms of inheritance in the F3 generation may be different from the F1 generation because significantly more changes were seen in the transcriptome.

**Electronic supplementary material:**

The online version of this article (doi:10.1186/s13058-017-0866-x) contains supplementary material, which is available to authorized users.

## Background

With 1.7 million new cases in 2012, breast cancer is the most common cancer in women worldwide, and the incidence is projected to continue growing [1]. However, only 5–10% of breast cancers are attributed to an inherited genetic cause [2, 3], and this leaves over 90% of breast cancers to be caused by some other factors. It is believed that environmental and lifestyle factors, such as diet, play a critical role in affecting breast cancer risk [4], and their effects on cancer susceptibility are likely to be mediated by epigenetic modifications [5].

Studies show that a Western dietary pattern, one that is higher in dietary fats, has been increasing around the world [6, 7] and may be linked to increased breast cancer risk [4]. Intake of dietary fats, especially saturated fatty acids and n-6 polyunsaturated fatty acids (n-6 PUFAs), has been climbing over the years [8], with intake of n-3 PUFAs declining [6]. A high-fat (HF) diet, especially one where n-6 PUFAs are high, increases estrogenic activities [9, 10] and is inflammatory [11, 12], which can stimulate breast cancer growth. Our laboratory and others have shown in animal models that maternal exposure to a HF diet during pregnancy increases female offspring’s mammary cancer risk [9, 13–16]. In addition, we found that maternal intake of an n-6 PUFA HF diet consumed before and during pregnancy increased mammary cancer risk of daughters (F1) and granddaughters (F2), but not of great-granddaughters (F3) [13], indicating multigenerational but not transgenerational inheritance [17]. Multigenerational inheritance of a trait caused by maternal exposures during pregnancy, such as an increased susceptibility to breast cancer, is seen in F1 and/or F2 but not in F3 generation offspring. If the trait is also seen in F3 generation, inheritance is transgenerational [18].

The lack of transgenerational inheritance in the offspring of HF diet-fed dams was puzzling because previously we and others discovered that several different exposures during pregnancy led to a transgenerational increase in mammary cancer risk and other adverse health effects in F1, F2, and F3 generations. Specifically, transgenerational inheritance of adult-onset diseases and abnormalities, such as mammary cancer [13], prostate disease [19], obesity [20], and autism-like phenotype [21], has been found in offspring of dams that were exposed to compounds such as ethinylestradiol [13], vinclozolin [19], dichlorodiphenyltrichloroethane (DDT) [20], and valproic acid [21] during pregnancy. Common to all these studies was that the maternal exposure took place after fetal implantation, whereas a HF diet that resulted in a multigenerational increase in mammary cancer risk was started before conception [13].

Mammals undergo two rounds of global DNA demethylation and remethylation during the embryonic and fetal periods [22]. It is possible that in our previous study, the reason why we did not see true transgenerational inheritance of increased breast cancer risk was due to introducing the HF exposure throughout the first round of DNA methylation erasure in the zygote and the subsequent remethylation, as well as during the second round of methylation erasure, which took place in primordial germ cells (PGCs), and the second remethylation, which reestablished epigenetic marks on mature germ cells [23]. Because PGCs originate from the posterior endoderm of the blastocyst, the second round of epigenetic programming may have been affected by the presence of the HF diet during the first round. To test a possibility that maternal HF intake induces a transgenerational inheritance of mammary cancer risk, we timed the HF exposure to start on gestational day (GD) 10. Pregnant women consume more dietary fats than nonpregnant women, and the increase takes place between the first and second trimesters [24, 25].

Our study shows that maternal intake of a HF diet between GDs 10 and 20 caused a transgenerational increase in mammary cancer risk tumors without any other intervening dietary exposures. Results from RNA sequencing (RNA-seq) analysis suggested that there was a difference in how mammary cancer risk was affected in F1 and F3 generation offspring by maternal HF intake (i.e., whether the exposure directly affected fetal somatic cells or the germline).

## Methods

### Breeding and dietary exposure

Male and female C57BL/6NTac mice were obtained from Taconic Biosciences (Germantown, NY, USA) and housed in standard rodent housing at constant temperature and humidity and with a 12-h/12-h light/dark cycle at Georgetown University’s Department of Comparative Medicine, in accordance with all institutional and federal regulations.

**Fig. 1:**
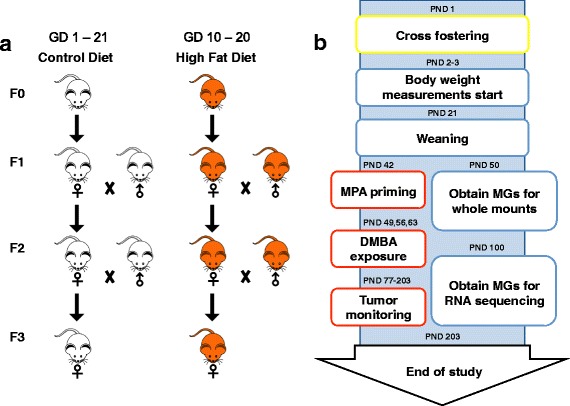
Transgenerational study design. **a** Pregnant C57BL/6NTac mice (F0) were fed either a high-fat (HF; *n* = 10) or control (CON; *n* = 10) diet. The HF diet was fed to dams from gestational day (GD) 10 to GD 20. All offspring were fed the CON diet after birth for the remainder of the study, including during pregnancies of F1 and F2 generation offspring. **b** All pups were cross-fostered at birth (postnatal day [PND] 1) to a CON mother to eliminate litter bias. Pups were weighed on PNDs 2 and 3 and weaned on PND 21. Tumorigenesis was initiated on PND 42 by priming female mice with medroxyprogesterone acetate (MPA; 15 mg/kg), followed by oral gavage of 7,12-dimethylbenz[a]anthracene (DMBA; 1 mg/dose/week) for 3 weeks. Tumorigenesis was monitored by palpation once per week starting 3 weeks after final DMBA administration up to 20 weeks post-DMBA. Mammary glands (MGs) and tumors were collected and processed from F1 and F3 offspring unexposed to DMBA on PND 50 for whole mounts and on PND 100 to perform RNA sequencing analysis

To generate F1 offspring (Fig. 1a), 7-week-old mice were mated by housing two females together with one male per cage. Upon mating, mice were randomly divided into two groups and fed a control AIN93G diet until pregnancy was verified by the presence of a mucus copulatory plug in the vaginal opening (GD 0). From GDs 10 to 20, the pregnant dams (F0) were divided into two groups and fed either a HF diet containing 41.1% energy from fat, 38.9% kcal of corn oil (CO), and 2.2% kcal of soybean oil (SBO) (Additional file 1: Table S1), or they were continued on the control diet (16.0% energy from fat, 13.7% kcal of CO, 2.2% kcal of SBO) (Additional file 1: Table S1). Because the length of pregnancy in these mice is 19–21 days, the HF exposure took place during the second and third mouse trimesters. The HF diet was made isocaloric with the control diet by replacing some carbohydrates with non-energy-containing cellulose (fiber). CO contains high levels of n-6 PUFAs. Both diets contained 10 g/kg SBO to ensure that pregnant dams received sufficient levels of n-3 PUFAs. All subsequent generations (F1–F3) were fed only the control diet.

To obtain F3 generation offspring, female F1 generation offspring of dams fed a HF diet during pregnancy were mated to F1 HF diet-fed males, respectively. No sibling matings were performed. The control group was mated similarly. Pregnant F1 dams of both groups were fed the CON diet. These steps were repeated with F2 offspring to obtain the F3 generation. The F2 generation was used for breeding purposes only; that is, no experiments were performed with or tissues collected from them.

### Mammary tumorigenesis

To induce mammary tumors (Fig. 1b), female C57BL6/NTac mice (*n* = 30 for F1 control, *n* = 30 for F1 HF; *n* = 20 for F3 control, and *n* = 25 for F3 HF offspring) were first primed with 15 mg/kg of medroxyprogesterone acetate (MPA; Greenstone, Peapack, NJ, USA) at postnatal day (PND) 42. One milligram of 7,12-dimethylbenz[a]anthracene (DMBA; Sigma-Aldrich, St. Louis, MO, USA) in 0.1 ml of CO was administered by oral gavage on PNDs 49, 56, and 63. After the last DMBA dose, tumor development was monitored by palpation once weekly for 20 weeks. If tumors were detected, their sizes were measured by calipers. The following endpoints were determined: incidence (number of mice with tumors), latency (time to first tumor), multiplicity (number of tumors per mouse), and burden (total tumor volume per mouse). The overall health of mice was monitored daily. A mouse was killed prior to the end of the tumor-monitoring period if it lost a significant amount of weight or if the tumor reached 10% of the mouse’s body weight. Mice killed for health reasons not pertaining to the study were excluded from the analysis. Tumor histopathology was assessed by a certified pathologist.

Kaplan-Meier survival curves were used to assess differences in tumor incidence between groups, followed by log-rank tests. Tumor latency and multiplicity differences were assessed by *t* test. Difference in tumor burden was assessed by repeated measures analysis of variance (ANOVA).

### Tissue collection

Fourth mammary glands were obtained on PND 50 and PND 100 from offspring not exposed to the carcinogen to assess changes in mammary gland morphology and transcriptome, respectively (Fig. 1b). At the end of the tumor-monitoring period, offspring in F1 and F3 generations were killed, and whole blood was collected via cardiac puncture and placed into serum gel separator tubes. We then collected mammary tumors, resected a portion for formalin-fixed, paraffin embedding, and flash-froze the remaining tissues in cryotubes in liquid nitrogen.

### Terminal end buds

Whole mounts were prepared according to an established protocol [26]. For that purpose, the left fourth abdominal mammary glands were stretched onto a slide, fixed, and stained with carmine aluminum solution. Slides were examined blindly under a microscope to determine the total number of terminal end buds (TEBs). These are the structures that give rise to malignant mammary tumors in mice and rats [27] and to similar structures in humans, called *terminal ductal lobular units*, which also are the sites of most breast cancers [28]. Differences in TEB numbers between the offspring of dams fed the HF or control diet were analyzed by *t* tests in both generations separately.

### RNA sequencing

To identify differentially expressed genes (DEGs) between offspring of dams exposed to the HF or control diet during pregnancy, RNA-seq was performed. Total RNA was extracted from the right fourth mammary glands obtained on PND 100 from F1 and F3 generation offspring that were not treated with DMBA by using the RNeasy Lipid Tissue Mini Kit (QIAGEN Sciences, Germantown, MD, USA) with on-column DNase digestion (QIAGEN Sciences) per the manufacturer’s protocol. The concentration and purity of the extracted RNA were determined by using a NanoDrop 1000 spectrophotometer (Thermo Scientific, Wilmington, DE, USA). The quality of the samples was assessed using a 2100 Bioanalyzer (Agilent Technologies, Santa Clara, CA, USA) for RNA integrity number (>7.0) and concentration (minimum 70 ng/μl). RNA-seq was performed by GENEWIZ (South Plainfield, NJ, USA). The HiSeq 2500 platform (Illumina, San Diego, CA, USA) was used for RNA-seq in a 1 × 50-bp single-read configuration in rapid run mode, with a total of at least 120 million reads per lane over 5 lanes with a read depth of at least 10 million reads per sample. A total of five control and three HF offspring in the F1 generation and four control and five HF offspring in the F3 generation were used for RNA-seq analysis.

Rsem was used to quantify transcript abundance using the mouse genome *Mus musculus* as a reference. With a *p* value cutoff of 0.05, we obtained 5620 DEGs for further analysis. We then selected those 48 DEGs that were seen in both F1 and F3 generation offspring, with the direction (up- or downregulation) of differential expression being similar in the two generations, and performed Ingenuity Pathway Analysis (IPA; QIAGEN Bioinformatics, Redwood City, CA, USA) to assess function of the DEGs. Knowledge-fused differential dependency network (KDDN) analysis was also performed on the 48 DEGs to assess transcriptional gene interaction unique to either control or HF offspring [29].

### Validation of RNA-seq data by quantitative real-time polymerase chain reaction

Of the 48 DEGs, we attempted to validate the expression of 13 genes that were selected on the basis of their reported association with breast cancer. RNA was extracted from the right fourth mammary glands of six control and six HF offspring of F1 generation and also of six control and six HF offspring of F3 generation using the RNeasy Lipid Tissue Mini Kit per the manufacturer’s protocol. These mice included those used for RNA-seq. The concentration, purity, and quality of RNA samples were assessed as described above. A quantity of 2 μg of RNA per sample was used to generate complementary DNA (cDNA) via reverse transcription using the High-Capacity cDNA Reverse Transcription Kit (Applied Biosystems, Foster City, CA, USA) and was run on a PTC-100 thermal cycler (Bio-Rad Laboratories, Hercules, CA, USA). Product cDNA was brought to a working concentration of 5 ng/μl and mixed with ABsolute QPCR, SYBR Green, ROX mix (Thermo Fisher Scientific, Waltham, MA, USA) and gene-specific forward and reverse primers. Primers used in quantitative polymerase chain reaction (qPCR) analysis were designed using IDT tool primer design (Integrated DNA Technologies, Coralville, IA, USA) (primer sequence provided in Additional file 2: Table S2). Real-time qPCR was carried out using a 7900HT Real-Time PCR system (Applied Biosystems). Expression of target genes was calculated by the relative standard curve method normalized to the housekeeping gene glyceraldehyde 3-phosphate dehydrogenase (*GAPDH*). Statistical differences between the control and F1 HF and F3 HF groups were assessed by one-way ANOVA.

## Results

### Effect of maternal HF intake on offspring mammary tumorigenesis

Maternal exposure to a HF diet during pregnancy did not affect the distribution of benign versus malignant mammary tumors among the offspring. Malignant mammary tumors were those that were either papillary or tubular adenocarcinomas or mammary carcinomas. Among the control offspring, 58.3% and 64.3% were malignant in the F1 and F3 generations, respectively (Additional file 3: Figure S1a), compared with 66.0% and 77.3% in the F1 and F3 generation HF offspring (Additional file 3: Figure S1b).

**Fig. 2:**
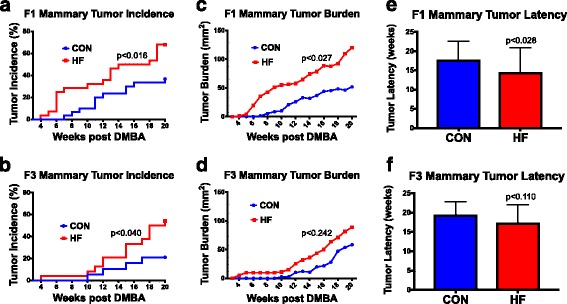
Transgenerational effect of maternal control (CON) or high-fat (HF) diet on offspring mammary tumorigenesis. Differences in mammary tumor incidence in (**a**) F1 (*p* < 0.016; CON, *n* = 30 mice; HF, *n* = 28 mice) and (**b**) F3 (*p* < 0.040; CON, *n* = 19 mice; HF, *n* = 24 mice) generation female offspring of dams fed either CON or HF diet during pregnancy. Differences in mammary tumor burden in (**c**) F1 (*p* < 0.027) and (**d**) F3 (*p* < 0.242) generation female offspring. Differences in mammary tumor latency in (**e**) F1 (*p* < 0.028) and (**f**) F3 (*p* < 0.110) generation female offspring. Mean ± SEM data are shown in **c**–**f**. *TEB* Terminal end bud

When we assessed differences in tumor incidence, we observed that only those with a malignant phenotype were included in the analysis. Female offspring exposed to the HF diet through a pregnant dam exhibited increased tumor incidence in F1 (Fig. 2a) (*p* < 0.016) and F3 (Fig. 2b) (*p* < 0.040) generations compared with control offspring. Mammary tumor burden was also increased in the F1 generation (Fig. 2c) (*p* < 0.027), but the increase failed to reach statistical significance in the F3 generation (Fig. 2d) (*p* < 0.242).

Maternal HF exposure during pregnancy induced earlier onset of mammary cancer in F1 generation (Fig. 2e) (*p* < 0.028) and had a similar trend in F3 generation offspring (Fig. 2f) (*p* < 0.110). Mammary tumor multiplicity was unaffected by maternal HF exposure (Additional file 4: Figure S2).

### Offspring mammary gland morphology

**Fig. 3:**
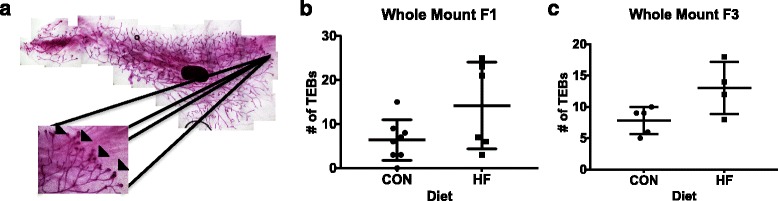
Effect of maternal control (CON) or high-fat (HF) diet exposure on offspring mammary gland development. **a** The left fourth mammary glands were obtained on postnatal day 50 for whole mounts. Terminal end buds, structures in the enlarged image indicated by the *arrows*, were counted for (**b**) F1 (*p* < 0.035; *n* = 8 for HF and *n* = 6 for CON) and (**c**) F3 (*p* < 0.023; *n* = 5 for HF and *n* = 4 for CON). Mean ± SEM data are shown. *DMBA* 7,12-Dimethylbenz[a]anthracene

Mammary gland morphology was assessed using whole mounts obtained from female offspring at PND 50. The number of TEBs (indicated by the *arrows* in Fig. 3a) was counted and found to be significantly higher in HF offspring for both F1 (Fig. 3b) (*p* < 0.035) and F3 generations (Fig. 3c) (*p* < 0.023).

### Differentially expressed genes in mammary glands of HF diet-exposed offspring

**Fig. 4:**
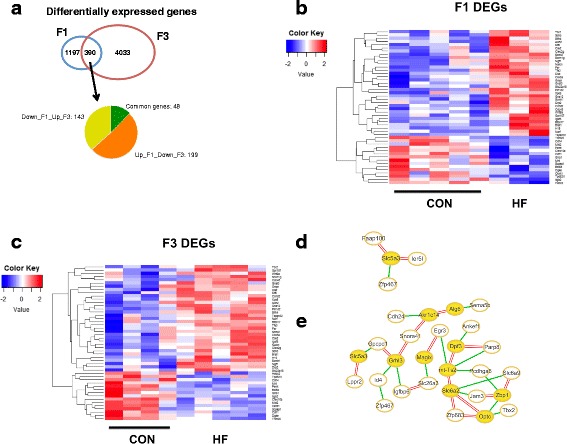
Differentially expressed genes (DEGs) in mammary glands of F1 and F3 generation offspring of dams fed either control (CON) or high-fat (HF) diet during pregnancy. **a** RNA-sequencing analysis identified 1587 DEGs in F1 and 4423 DEGs in F3 generation mammary glands obtained on postnatal day 100 from mice not exposed to 7,12-dimethylbenz[a]anthracene (*n* = 5 CON and *n* = 3 HF offspring in F1 generation, and *n* = 4 CON and *n* = 5 HF offspring in F3 generation). A total of 390 common DEGs were found in the F1 and F3 generations, with 48 regulated in the same direction in both generations. Heat map of common 48 DEGs in (**b**) F1 mammary glands and (**c**) F3 mammary glands. Knowledge-fused differential dependency networks cluster map of nodes uniquely connected to different sets of genes in HF or CON offspring in (**d**) F1 or (**e**) F3 generation. *Yellow ovals* indicate nodes. *Single-lined green connections* indicate gene interactions in HF offspring. *Double-lined red connections* indicate gene interaction in CON offspring

To elucidate the potential differences in mammary cancer risk in the F1 and F3 generation offspring of HF diet-fed dams, RNA-seq analysis was performed on normal mammary glands. In the F1 generation, 1587 DEGs were identified, and in the F3 generation, 4423 DEGs were seen (Fig. 4a). Of these, 390 were the same genes in both F1 and F3 HF offspring. However, only 48 of the DEGs were altered in the same direction (up- or downregulated) in both generations. Heat maps of these genes are shown in Fig. 4b and c and Additional file 5: Table S3.

IPA indicated that the top pathways that were different between HF and control offspring in both F1 and F3 generations were related to vitamin D receptor/retinoid X receptor (VDR/RXR) activation, phosphatase and tensin homolog (*PTEN*) signaling, farnesoid X receptor/RXR (FXR/RXR) activation, hereditary breast cancer signaling, and Notch signaling (Additional file 6: Table S4). Top upstream regulators of these pathways were interferon regulatory factors 3 and 7 (*IRF3* and *IRF7*, respectively) linked to interferon and macrophage regulation [30], and delta like canonical ligand 3 (*DLL3*), Jagged 1 (*JAG1*), and mesogenin 1 (*MSGN1*), which all are linked to Notch signaling [31, 32] (Additional file 6: Table S4). Top diseases and biofunctions involved development, cellular functions, cancer and tumor morphologies, and inflammatory responses (Additional file 7: Table S5).

KDDN analysis (Fig. 4d and e, Table 1) performed to identify unique gene signaling interactions present in either the HF or control offspring highlighted the following ten genes as nodes: *ALG6*, *DPF3*, *GRHL3*, *MAGIX*, *MT-TS2*, and *OPTC* (full gene names provided in Abbreviations section below; upregulated in HF offspring), as well as *AKR1C14*, *SLC5A3*, *SLC6A2*, and *ZBP1* (downregulated in HF offspring). These nodes had different signaling connections in HF and CON offspring. The node genes in the offspring of HF diet-fed dams were linked to changes in genes that are indicative of increased cancer risk (downregulation of *ANKEF1*, *IGFBP6*; upregulation of *SEMA5B*), increased resistance to cancer treatments (upregulation of *EGR3*, *SLC26A3*), poor cancer prognosis (upregulation of *ID4*, *JAM3*, *TBX2*), increased risk of metastasis (upregulation of *GPCPD1*) and impaired anticancer immune response (downregulation of *ZBP1*, upregulation of *EGR3*) (Additional file 8: reference table). In contrast, the ten node genes in the mammary glands of the offspring of dams fed the CON diet were linked to changes in the expression of genes indicative of reduced cancer risk (downregulation of *DPF3*, *SNORA41*) and improved immune functions (upregulation of *ZBP1*, *ZFP683*; downregulation of *EGR3*). These differences potentially play a regulatory role in causing transgenerational inheritance of increased mammary cancer risk in the offspring of dams fed the HF diet during pregnancy.

**Table 1:** Upregulated and downregulated node genes in normal mammary glands of F1 and F3 generation mouse offspring of dams fed a high-fat diet and their unique signaling connections identified via knowledge-fused differential dependency network analysis^a^

Upregulated gene (function)	Connections in HF offspring	Consequence	Connections in control offspring	Consequence
*Alg6* Encodes glucosyltransferase, critical role in N-glycosylation [1]	Sema5b↑	*O*-glycosylation of thrombospondin type 1 repeat domain-containing proteins; implicated in clear cell renal cell carcinoma [17] as well as other age-related diseases, including cancer [18]	Akr1c14↑	3α-Hydroxysteroid dehydrogenase enzyme; catalyzes conversion of potent testosterones into less potent forms [8]
*Dpf3* Chromatin remodeling; associated with increased breast cancer risk, tumor size, earlier onset of disease, and lymph node metastases [2]; also implicated in chronic lymphocytic leukemia through STAT5 regulation [3]	Ankef1↓	Calcium ion binding; found as significant predictor of prostate cancer in GWAS SNPs study [19]	Parp8↓	Upregulated in acute leukemia [20]; catalyzes posttranslational modification of protein by addition of ADP-ribose moieties; contributes to survival of injured proliferating cells [21]
			Mt-Ts2↑	RNA gene affiliated with noncoding RNA class; possible association with mitochondrial disorders [9]
*Grhl3* Regulator of developmental processes; overexpression increases cell migration and invasion by downregulating E-cadherin [4]; strongly implicated in breast cancer [5]	Id4↑	A lineage-dependent proto-oncogene that is overexpressed and amplified in a subset of basal-like breast cancers and confers a poor prognosis [22]; suppresses *BRCA1* [23, 24]	Gpcpd1↓	Downregulation reduces migration capacity of tumor cells and is a prognostic indicator of good outcome in endometrial and ovarian cancers [25]
	Igfbp6↓	IGF-1-binding protein; lower expression in malignant breast cancer than in benign tumors [26]; also overexpressed in lung cancer and rhabdomyosarcomas [27]	Snora41↓	Long noncoding RNA linked to embryonic stem cell differentiation [28]; upregulated in lung cancer [29]
*Magix* Function not known	Slc26a3↑	Glycoprotein, a marker of chemoresistance in ER^+^ breast cancer [30]	Egr3↓	Mediates E2-induced breast cancer metastasis [31]; upregulated in endocrine-resistant breast cancers [32]; increases aromatase in breast tissue [33]; maintains tumor immunosuppression [34]
*Optc* Extracellular matrix glycoprotein [6], found translocated to nucleus in chronic lymphocytic leukemia cells [7]	Tbx2↑	Regulator of developmental processes; increased expression predicts poor prognosis for many cancer types, including head and neck cancers [35], gastric cancer [36], and breast cancer [37]; contributes to drug resistance in breast cancer [38]	Slc6a2↑	Induces norepinephrine uptake [11]; upregulation lowers risk of pancreatic ductal adenocarcinoma [12] and non-small-cell lung cancer [13]
	Zbp1↓	Activator of innate immune response [14, 15] with potential to promote effective antitumor CD8^+^ T-cell immunity [16]	Zfp683↑	Essential for formation of mature thymic natural killer cells [39]
Downregulated gene (function)	Connections in HF	Consequence	Connections in CON	Consequence
*Akr1c14* 3α-hydroxysteroid dehydrogenase enzyme; catalyzes conversion of potent testosterones into less potent forms [8]	Cdh24↑	Induces cell adhesion; mutation target in cancers with microsatellite instability, particularly gastric and colorectal cancers [40]	Alg6↓	Encodes glucosyltransferase, critical role in N-glycosylation [1]
			Snora41↓	Long noncoding RNA linked to embryonic stem cell differentiation [28]; upregulated in lung cancer [29]
*Mt-Ts2* RNA gene affiliated with noncoding RNA class; possible association with mitochondrial disorders [9]	Egr3↑	Mediates E2-induced breast cancer metastasis [31]; upregulated in endocrine-resistant breast cancers [32]; increases aromatase in breast tissue [33]; maintains tumor immunosuppression [34]	Dpf3↓	Polymorphisms in 5′ region associated with increased breast cancer development
	Parp8↑	Upregulated in acute leukemia [20]; catalyzes posttranslational modification of protein by addition of ADP-ribose moieties, contributes to survival of injured proliferating cells [21]	Zbp1↑	Activator of innate immune response [14, 15] with potential to promote effective antitumor CD8^+^ T-lymphocyte immunity [16]
	Pcdhga8↑	Establishes cell-cell connections [41]		
*Slc5a3* Transporter; downregulation impairs cellular functions by causing accumulation of myoinositol, downregulated in type 2 diabetes [10]	Gpcpd1↑	Decreased expression increases the migration capacity of tumor cells and worsens prognosis of endometrial and ovarian cancers [25]	Ier5l↓	Immediate early response gene; may mediate actions on PUFAs [45]
	Zfp467↑	Promotes adipocyte differentiation and inhibits osteoblast differentiation [43]	Faap100↓	Required for E3 ligase function [44]
			Lppr2↓	Peptide-ligand binding, GPCR signaling [42]
*Slc6a2* Norepinephrine uptake regulator [11]; downregulation increases risk of pancreatic ductal adenocarcinoma [12] and non-small-cell lung cancer [13]	Jam3↑	Cell-cell adhesion; upregulation associated with poor prognosis for non-small-cell lung cancer [46] and gastric cancer [47]; promotes ovarian tumors in mice [48]	Optc↓	Extracellular matrix glycoprotein [6], found translocated to nucleus in chronic lymphocytic leukemia cells [7]
	Slc6a9↑	Transporter that inhibits glycine signaling [49]; solute carrier 6 family is implicated in many cancers [50]	Zbp1↑	Activator of innate immune response [14, 15] with potential to promote effective antitumor CD8^+^ T-cell immunity [16]
			Zfp683↑	Essential for formation of mature thymic natural killer cells [39]
*Zbp1* Activator of innate immune response [14, 15] with potential to promote effective antitumor CD8^+^ T-cell immunity [16]	Pcdhga8↑	Establishes cell-cell connections [41]	Slc6a2↑	Induces norepinephrine uptake [11]; upregulation lowers risk of pancreatic ductal adenocarcinoma [12] and non-small-cell lung cancer [13]
	Optc↑	Extracellular matrix glycoprotein [6] found translocated to nucleus in chronic lymphocytic leukemia cells [7]	Jam3↓	Cell-cell adhesion; downregulation linked to better prognosis for non-small-cell lung cancer [46] and gastric cancer [47]; upregulation promotes ovarian tumor in murine model [48]
			Mt-Ts2↑	RNA gene, affiliated with noncoding RNA class, possible association with mitochondrial disorders [9]
			Slc6a9↓	Transporter that inhibits glycine signaling [49]; solute carrier 6 family is implicated in many cancers [50]

*Abbreviations: Akr1c14* Aldo-keto reductase family 1, member C14, *Alg6* α-1,3-Glucosyltransferase, *Ankef1* Ankyrin repeat and EF-hand domain containing 1, *Dpf3* Double PHD fingers 3, *E2* 17-β-Estradiol, *Egr3* Early growth response 3, *ER* Estrogen receptor, *Gpcpd1* Glycerophosphocholine phosphodiesterase 1, *GPCR* G protein-coupled receptor, *Grhl3* Grainyhead like transcription factor 3, *GWAS* Genome-wide association study, *HF* High fat, *Id4* DNA-binding protein inhibitor ID-4, *IGF-1* Insulin-like growth factor 1, *Igfbp6* Insulin-like growth factor binding protein 6, *Jam3* Junctional adhesion molecule 3, *Magix* MAGI family member, X-linked, *Mt-Ts2* Mitochondrially encoded transfer RNA serine 2 (AGU/C), *Optc* Opticin, *Pcdhga8* Protocadherin gamma subfamily A, 8, *PUFA* Polyunsaturated fatty acid, *Sema5b* Semaphorin 5B, *Slc26a3* Solute carrier family 26 member 3, *Slc5a3* Solute carrier family 5 member 3, *Slc6a2* Solute carrier family 6 member 2, *Snora41* Small nucleolar RNA, H/ACA box 41, *SNP* Single-nucleotide polymorphism, *STAT5* Signal transducer and activator of transcription 5, *Tbx2* T-box 2, *Zbp1* Z-DNA binding protein 1, *Zfp683* Zinc finger protein 683

^a^ Connection of the node genes in control offspring are also shown

### Verification of differential gene expression

**Fig. 5:**
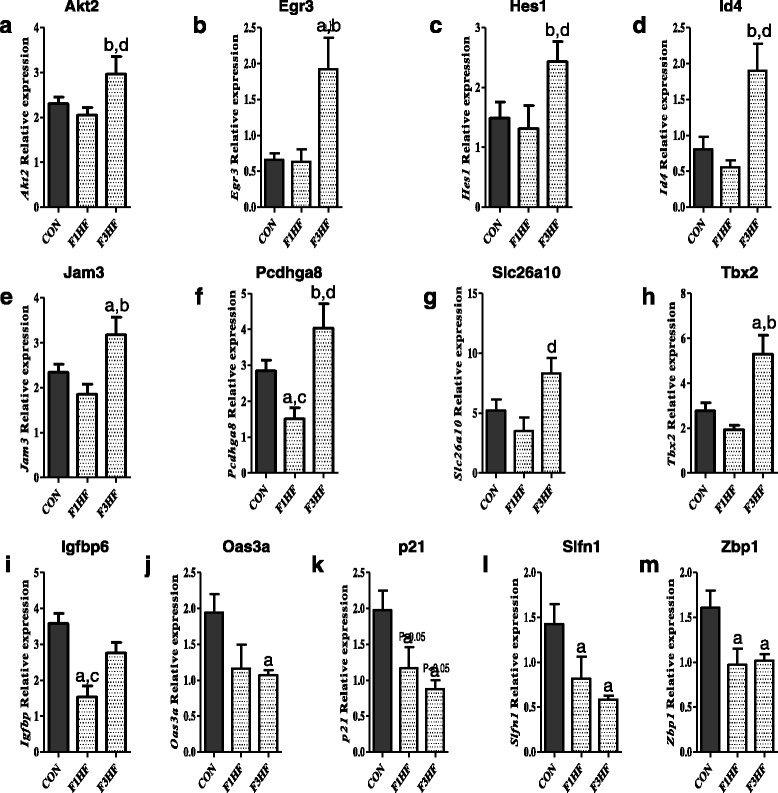
Verification of differential gene expression. Validation by quantitative real-time polymerase chain reaction of the following 13 differentially expressed genes identified in RNA-sequencing analysis: (**a**) *Akt2*, (**b**) *Egr3*, (**c**) *Hes1*, (**d**) *Id4*, (**e**) *Jam3*, (**f**) *Pcdhga8*, (**g**) *Slc26a10*, (**h**) *Tbx2*, (**i**) *Igfbp6*, (**j**) *Oas3a*, (**k**) *p21*, (**l**) *Slfn1*, and (**m**) *Zbp1* (*p* < 0.05, **a** different from control diet [CON], **b** different from F1 high-fat (HF) diet, **c** different from F3 HF; *p* < 0.06, **d** marginally different from CON). We used fourth mammary glands obtained on postnatal day 100 from six CON and six HF offspring in F1 generation, as well as from six control and six HF offspring in F3 generation for the analysis. Mean ± SEM data are shown. *Akt2* Serine/threonine kinase 2, CON Control diet, *Egr3* Early growth response 3, *Hes1* Hairy and enhancer of split-1, *HF* High fat, *Id4* DNA-binding protein inhibitor ID-4, *Igfbp6* Insulin-like growth factor binding protein 6, *Jam3* Junctional adhesion molecule 3, *Oas3* 2′-5′-Oligoadenylate synthetase 3, *Pcdhga8* Protocadherin gamma subfamily A, 8, *Slc26a10* Solute carrier family 26 member 10, *Slfn1* Schlafen 1, *Zbp1*, Z-DNA binding protein 1

Quantitative real-time polymerase chain reaction (qRT-PCR) analysis indicated that all 13 genes differentially expressed in the RNA-seq dataset between offspring of HF and control diet-fed dams were validated in the F3 generation (Fig. 5). However, among the F1 generation, none of the eight upregulated genes were validated: *AKT2*, *EGR3*, *HES1*, *ID4*, *JAM3*, *PCDHGA8*, *SLC26A10*, and *TBX2* (Fig. 5a–h). Of the five downregulated genes (*IGFBP6*, *OAS3a*, *P21*, *SLFN1*, and *ZBP1*) (Fig. 5i–m), four were significantly and one was nonsignificantly downregulated in both the F1 (*OAS3a* was not significant) and F3 (*IGFBP6* was not significant) generations.

## Discussion

We found that maternal intake of a HF n-6 PUFA diet, starting on GD 10 during the second half of pregnancy and continuing until the end of pregnancy, increased estrogen receptor-positive (ER^+^) mammary cancer risk in F1 and F3 generation mouse offspring. Transgenerational inheritance likely requires that epigenetic changes induced by maternal exposures during pregnancy persist in germ cells. This possibility has been debatable because when F1 generation germ cells become fertilized, parental DNA methylation patterns are erased in the zygote [17, 22, 23]. However, a growing number of studies indicate that some genes in preimplantation zygotes can escape the complete loss of methylation marks that were established during reprogramming events of germ cells [32, 33]. Further, it has been shown that changes in histone marks in the preconception oocytes can be transmitted across generations [34]. Results of our previous studies, as well as results of multiple other studies, indicate that various maternal exposures after the first week of gestation, such as endocrine disruptors ethinylestradiol (EE2) [13], 2,3,7,8-tetrachlorodibenzo-*p*-dioxin (TCDD) [35], vinclozolin [19], or DDT in rats [20], or bisphenol A [36], cause transgenerational alterations seen in F3 generation offspring.

Why, then, is the F3 generation not affected [13] if the exposure starts before conception and involves both the preimplantation period and the period when PGCs travel to the genital ridge [22, 23]? It is possible that although F1 generation PGCs are affected directly by the in utero HF environment in both our previous study and the present study, the changes can be transgenerationally inherited only if the somatic cells in the blastocyst giving rise to PGCs were not also affected. If true, maternal exposures that start before conception and continue through pregnancy can have multigenerational effects only involving the F1 and F2 generations, but not the F3 generation.

To determine if similar changes occur in gene transcription in F1 and F3 generations, when they are caused by a direct exposure in F1 generation or inherited in F3 generation through the germline, we performed RNA-seq analysis using normal mammary glands unexposed to the carcinogen DMBA, which were obtained from 100-day-old offspring of HF and control diet-fed dams. Surprisingly, over three times more DEGs were seen in the F3 than in the F1 generation mammary glands. Other groups have also reported greater gene transcription differences in F3 than F1 generation offspring. Ma et al. [35] found that maternal exposure to TCDD between GDs 8 and 14 resulted in a greater increase in both messenger RNA and protein expression of the imprinted gene insulin-like growth factor 2 (*IGF2*) in the rat liver of F3 than in F1 offspring. The increase was not caused by differences in DNA methylation patterns, which appeared similar in F1 and F3 generation offspring [35]. Consistent with these data, the differences in DNA methylation that we found earlier in F1 and F3 generations between control and maternal EE2 exposure groups were similar [13]. Thus, mechanisms other than changes in DNA methylation likely explain the increase in DEGs in the F3 generation offspring compared with F1 generation offspring.

From among all the DEGs found in our RNA-seq analysis, we selected the ones that were similarly altered in both F1 and F3 generation offspring of dams fed a HF diet during pregnancy compared with their controls for further analysis, because there possibly exists a connection between these 48 DEGs and the transgenerational inheritance of increased breast cancer risk. According to IPA, top biological functions of the DEGs were (1) development (cellular, embryonic, organ, organismal, and tissue), (2) cellular functions (cell cycle, proliferation, and morphology), (3) cancer and tumor morphology, and (4) inflammatory response (Additional file 7: Table S5). Considering that the main component of the HF diet was CO, a source of n-6 PUFAs, it is not surprising to find inflammatory response on this list of affected biofunctions. n-6 PUFAs and their arachidonic acid-derived eicosanoids are considered proinflammatory and associated with many inflammatory diseases, such as various types of cancer [11].

Five top upstream regulators of the 48 DEGs were *DLL3* and *JAG1* (Notch ligands) [37]; *MSGN1* (transcriptional activator of a Notch signaling program) [32]; and *IRF3* and *IRF7*, which are both key transcriptional regulators of interferons and macrophages [30]. Notch signaling may also regulate IRF activity [38]. These results suggest that Notch signaling may be altered in the offspring by maternal HF intake. Notch signaling was also among the five top pathways altered in the offspring of HF diet-fed dams, in addition to VDR/RXR and FXR/RXR activation, hereditary breast cancer signaling, and *PTEN* signaling. Researchers in earlier studies have found that maternal HF diet intake increases offspring Notch signaling in the hippocampus [39] and neural stem cells in mice [40]. Because the Notch pathway regulates stem cell maintenance, cell fate specification, differentiation, proliferation, motility, and survival during embryonic development and in cancers [41], our findings indicate that upregulation of Notch signaling in the normal mammary glands in F1 and F3 generation offspring of HF diet-fed dams may contribute to their increased mammary cancer risk.

To further characterize the functional roles of the DEGs, the networks hosted by these genes were constructed using KDDN analysis [29]. KDDN analysis identified differential connections among transcription factors that exist only in the mammary glands of offspring of HF diet-fed dams or only in the control offspring. In the HF group, genes identified as connected to the node genes were related to poor prognosis (*SEMA5B*, *ID4*, *TBX2*, *GRHL3*, *DPF3*), increased cellular proliferation and migration (*GRHL3*, *PARP8*, *JAM3*), and altered immune response (*ZBP1*, *EGR3*). As an example, the KDDN analysis indicated that upregulated grainyhead like transcription factor 3 (*GRHL3*) interacted with inhibitor of DNA binding 4 (*ID4*) and insulin-like growth factor binding protein 6 (*IGFBP6*) in the HF group, whereas its downregulation in the control group interacted with glycerophosphocholine phosphodiesterase 1 (*GPCPD1*) and small nucleolar RNA, H/ACA Box 41 (*SNORA41*). Upregulation of *GRHL3* is strongly implicated in breast cancer [42], possibly by increasing the epithelial-mesenchymal transition [43]. *ID4*, upregulated in the offspring of HF diet-fed dams, is associated with poor prognosis of breast cancer, inhibits *BRCA1* function in basal-like breast cancer [44], and promotes chemoresistance [45]. *IGFBP6* was downregulated in HF offspring, and it acts as a tumor suppressor [46]. The interactions of downregulated *GRHL3* in the control offspring with downregulated *GPCPD1* and *SNORA41* are indicative of good cancer prognosis [47, 48]. Changes in the expression of these genes and their interaction with each other suggest that maternal HF n-6 PUFA diet may not only lead to a transgenerational increase in breast cancer risk but also increase breast cancer mortality. This conclusion is consistent with our recent finding that maternal EE2 exposure during pregnancy increased resistance to antiestrogen therapy of ER^+^ mammary tumors in the offspring [49].

## Conclusions

Our findings indicate that consuming a HF n-6 PUFA diet between GDs 10 and 20 during pregnancy causes a transgenerational increase in mammary cancer risk in mice. We also observed over three times more changes in the mammary gland transcriptome in F3 than in F1 generation offspring of HF diet-fed dams, suggesting that germline inheritance of increased mammary cancer risk may involve additional pathways to those altered in the adult mammary gland by a direct exposure of the fetal somatic cells.

## Additional files

Additional file 1: **Table S1.** Nutritional content of control (modified AIN93-G) and high-fat n-6 PUFA diets fed to pregnant mouse dams. (DOCX 15 kb)

Additional file 2: **Table S2.** Primer sequences used in this study. (DOCX 77 kb)

Additional file 3: **Figure S1.** Tumor histopathology of all tumors collected from F1 and F3 generation offspring of dams exposed to either control (CON) or high-fat (HF) diet during pregnancy. **a** Tumor status of CON offspring. **b** Tumor status of HF offspring. (PDF 129 kb)

Additional file 4: **Figure S2.** Mammary tumor multiplicity was not altered between (**a**) F1 control (CON; *n* = 30 mice) and high-fat (HF; *n* = 29 mice) offspring or (**b**) F3 CON (*n* = 19 mice) and HF (*n* = 24 mice) generation offspring. (PDF 54 kb)

Additional file 5: **Table S3.** Common differentially expressed genes in the mammary glands of F1 and F3 generation offspring of dams fed high-fat n-6 PUFA diet during pregnancy, compared with control mice. (DOCX 104 kb)

Additional file 6: **Table S4.** Top differentially expressed pathways and predicted upstream regulators in F1 and F3 offspring of control or high-fat fed dams, identified in Ingenuity Pathway Analysis. (DOCX 47 kb)

Additional file 7: **Table S5.** Top differentially expressed diseases and biofunctions between F1 and F3 offspring of dams fed control or high-fat diet during pregnancy, identified using Ingenuity Pathway Analysis. (DOCX 65 kb)

Additional file 8: Supplementary reference list for KDDN. (DOCX 102 kb)

## Abbreviations

AKR1C14: Aldo-keto reductase family 1, member C14
AKT2: Serine/threonine kinase 2
ALG6: α-1,3-Glucosyltransferase
ANKEF1: Ankyrin repeat and EF-hand domain containing 1
ANOVA: Analysis of variance
cDNA: Complementary DNA
CO: Corn oil
CON: Control
DDT: Dichlorodiphenyltrichloroethane
DEG: Differentially expressed gene
DLL3: Delta like canonical Notch ligand 3
DMBA: 7,12-Dimethylbenz[a]anthracene
DPF3: Double PHD fingers 3
E2: 17-β-Estradiol
EE2: Ethinylestradiol
EGR3: Early growth response 3
ER: Estrogen receptor
FXR/RXR: Farnesoid X receptor/retinoid X receptor
GAPDH: Glyceraldehyde 3-phosphate dehydrogenase
GD: Gestational day
GPCPD1: Glycerophosphocholine phosphodiesterase 1
GPCR: G protein-coupled receptor
GRHL3: Grainyhead like transcription factor 3
GWAS: Genome-wide association study
HES1: Hairy and enhancer of split-1
HF: High fat
ID4: DNA-binding protein inhibitor ID-4
IGF-1: Insulin-like growth factor 1
IGF2: Insulin-like growth factor 2
IGFBP6: Insulin-like growth factor binding protein 6
IPA: Ingenuity Pathway Analysis
IRF3: Interferon regulatory factor 3
IRF7: Interferon regulatory factor 7
JAG1: Jagged 1
JAM3: Junctional adhesion molecule 3
KDDN: Knowledge-fused differential dependency network
MAGIX: MAGI family member, X-linked
MPA: Medroxyprogesterone acetate
MSGN1: Mesogenin 1
MT-TS2: Mitochondrially encoded transfer RNA serine 2 (AGU/C)
OAS3: 2′-5′-Oligoadenylate synthetase 3
OPTC: Opticin
P21: Cyclin-dependent kinase inhibitor 1
PCDHGA8: Protocadherin gamma subfamily A, 8
PGC: Primordial germ cell
PND: Postnatal day
PTEN: Phosphatase and tensin homolog
PUFA: Polyunsaturated fatty acid
qPCR: Quantitative polymerase chain reaction
qRT-PCR: Quantitative real-time polymerase chain reaction
RNA-seq: RNA sequencing
SBO: Soybean oil
SEMA5B: Semaphorin 5B
SFA: Saturated fatty acid
SLC26A10: Solute carrier family 26 member 10
SLC26A3: Solute carrier family 26 member 3
SLC5A3: Solute carrier family 5 member 3
SLC6A2: Solute carrier family 6 member 2
SLFN1: Schlafen 1
SNORA41: Small nucleolar RNA, H/ACA box 41
SNP: Single-nucleotide polymorphism
STAT5: Signal transducer and activator of transcription 5
TBX2: T-box 2
TCDD: 2,3,7,8-tetrachlorodibenzo-*p*-dioxin
TEB: Terminal end bud
VDR/RXR: Vitamin D receptor/retinoid X receptor
ZBP1: Z-DNA binding protein 1
ZFP683: Zinc finger protein 683
