# Supplementary material for: Maternal intake of high n-6 polyunsaturated fatty acid diet during pregnancy causes transgenerational increase in mammary cancer risk in mice
Source: Breast Cancer Res. 2017 Jul 3;19:77. doi: 10.1186/s13058-017-0866-x (PMC5494892; doi:10.1186/s13058-017-0866-x)
Supplement: Supplementary file 1 — Table S1. Nutritional content of control (modified AIN93-G) and high-fat n-6 PUFA diets fed to pregnant mouse dams. (DOCX 15 kb) [file 13058_2017_866_MOESM1_ESM.docx]

**Table S1.** Nutritional content of control (modified AIN93-G) and high fat n-6 PUFA diets fed to pregnant mouse dams.

| **Diet** | **Control (CON)**  **(TD.08819)** | | | |  |  | **High Fat (HF)**  **(TD.120672)** | | | |
| --- | --- | --- | --- | --- | --- | --- | --- | --- | --- | --- |
| **Energy from fat**  **Energy density** | 17.2%  3.8 Kcal/g | | |  | |  | 42.7%  4.0 Kcal/g | | | |
|  |  |  |  |  | |  |  |  | |  |
| **Formula** |  | **g/Kg** |  | **%Kcal** | |  |  | **g/Kg** | **%Kcal** | |
| Casein |  | 200.000 |  | 19 | |  |  | 215.000 | 19 | |
| L-Cystine |  | 3.000 |  | 0.3 | |  |  | 3.000 | 0.3 | |
| Corn Starch |  | 397.386 |  | 38 | |  |  | 162.386 | 14.4 | |
| Maltodextrin |  | 132.000 |  | 13.3 | |  |  | 132.000 | 12.4 | |
| Sucrose |  | 100.000 |  | 10.8 | |  |  | 100.000 | 9.9 | |
| Corn Oil |  | 60.000 |  | 14.4 | |  |  | 180.000 | 40 | |
| Soybean Oil |  | 10.000 |  | 2.4 | |  |  | 10.000 | 2.2 | |
| Cellulose |  | 50.000 |  | 0 | |  |  | 150.000 | 0 | |
| AIN-93G-MX |  | 35.000 |  | 0.8 | |  |  | 35.000 | 0.8 | |
| AIN-93-VX |  | 10.000 |  | 1 | |  |  | 10.000 | 1 | |
| Choline Bitartrate |  | 2.500 |  | 0 | |  |  | 2.500 | 0 | |
| TBHQ, antioxidant |  | 0.014 |  | 0 | |  |  | 0.014 | 0 | |
| Yellow food color |  | 0.100 |  | 0 | |  |  |  | 0 | |
| Orange food color |  |  |  |  | |  |  | 0.100 |  | |
