# Supplementary material for: Maternal intake of high n-6 polyunsaturated fatty acid diet during pregnancy causes transgenerational increase in mammary cancer risk in mice
Source: Breast Cancer Res. 2017 Jul 3;19:77. doi: 10.1186/s13058-017-0866-x (PMC5494892; doi:10.1186/s13058-017-0866-x)
Supplement: Supplementary file 2 — Table S2. Primer sequences used in this study. (DOCX 77 kb) [file 13058_2017_866_MOESM2_ESM.docx]

**Table S2.** Primer sequences used in study

| **Gene ID** | **Sequence** |
| --- | --- |
| mAKT2 F | TGCCATTCTACAACCAGGAC |
| mAKT2 R | CCTCTGCTTTGGGTCCTTC |
|  |  |
| mEGR3 F | AATCTGTACCCCGAGGAGAT |
| mEGR3 R | ATCACATTCTCTCTCCCACCG |
|  |  |
| mHES1 F | GTCTACACCAGCAACAGTGG |
| mHES1 R | GACTTTACGGGTAGCAGTGG |
|  |  |
| mID4 F | TGAACAAGCAGGGTGACAG |
| mID4 R | CGGTGGCTTGTTTCTCTTAATTTC |
|  |  |
| mJAM3 F | AAGATTCAAGGAGACCTGGCA |
| mJAM3 R | TAGAGCAACGACCTCACAGC |
|  |  |
| mPCDHGA8 F | GACTCTCCAGCTTTCCGTAAG |
| mPCDHGA8 R | CCATGCCTGTGTTGATTCTTG |
|  |  |
| mSLC26A10 F | CTCAGTGGAATCGAAAGGGAG |
| mSLC26A10 R | CGATAGAAAGGTGGACAGGAC |
|  |  |
| mTBX2 F | CACAAACTGAAGCTGACCAAC |
| mTBX2 R | GAAGACATAGGTGCGGAAGG |
|  |  |
| mIGFBP6 F | GCTCTATGTGCCAAACTGTG |
| mIGFBP6 R | TGAGTGCTTCCTTGACCATC |
|  |  |
| mOAS3 F | GCCTGCTTTTGATGCTGTG |
| mOAS3 R | GGAGGGCAAGTGTTTATGAAG |
|  |  |
| mP21 F | CTGTCTTGGACTCTGGTGTCTGA |
| mP21 R | CCAATCTGCGCTTGGAGTGA |
|  |  |
| mSLFN1 F | CATAGAGGAATGGATCAAGCTCC |
| mSLFN1 R | AAACCCTTCCAACATCCCC |
|  |  |
| mZBP1 F | GGACAGACGTGGAAGATCTAC |
| mZBP1 R | ATGGAGATGTGGCTGTTGG |
|  |  |
