# Supplementary material for: Maternal intake of high n-6 polyunsaturated fatty acid diet during pregnancy causes transgenerational increase in mammary cancer risk in mice
Source: Breast Cancer Res. 2017 Jul 3;19:77. doi: 10.1186/s13058-017-0866-x (PMC5494892; doi:10.1186/s13058-017-0866-x)
Supplement: Supplementary file 3 — Figure S1. Tumor histopathology of all tumors collected from F1 and F3 generation offspring of dams exposed to either control (CON) or high-fat (HF) diet during pregnancy. a Tumor status of CON offspring. b Tumor status of HF offspring. (PDF 129 kb) [file 13058_2017_866_MOESM3_ESM.pdf]

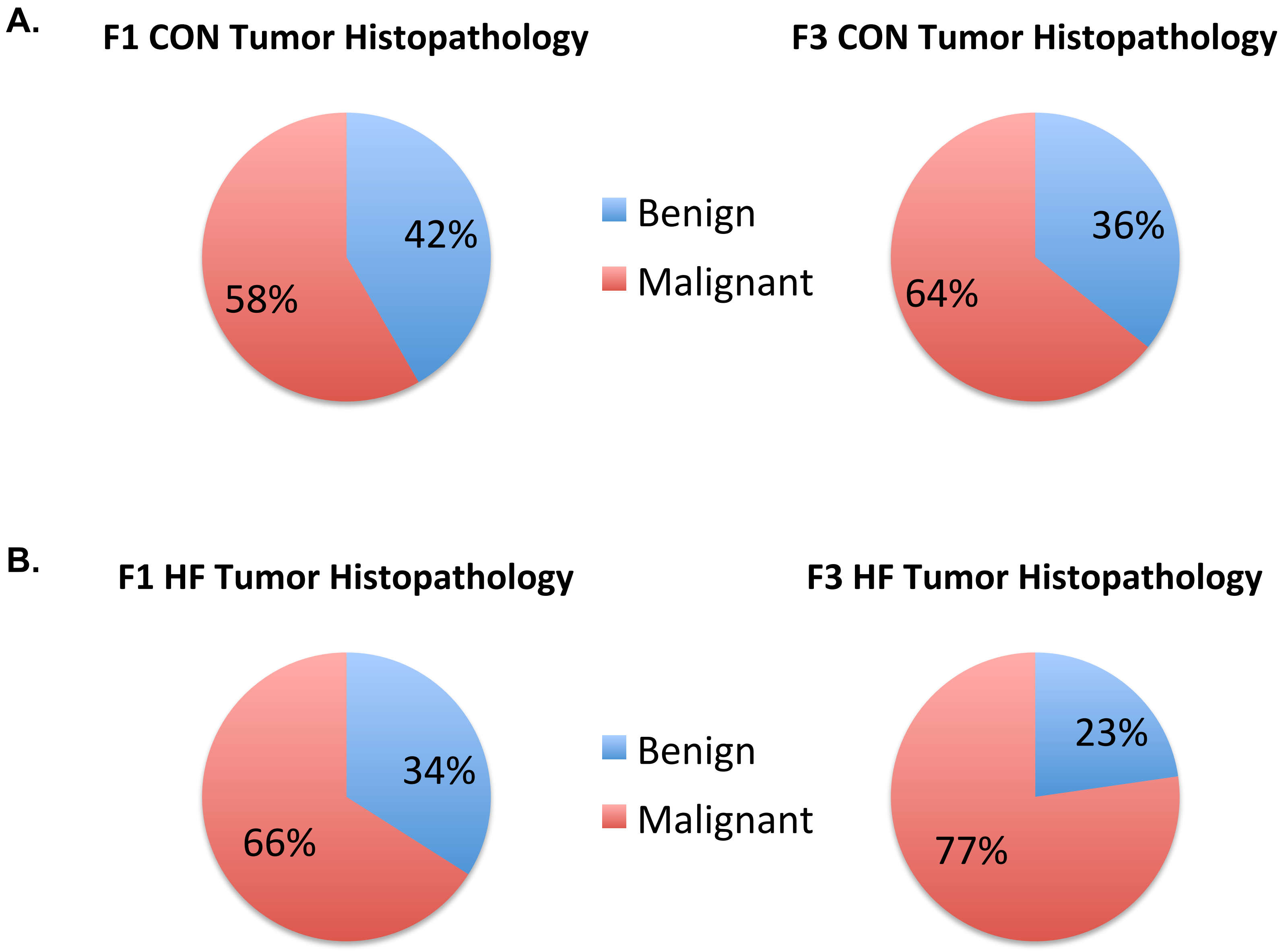
A.
F1 CON Tumor Histopathology
42%
58%
Benign
Malignant
F3 CON Tumor Histopathology
36%
64%
B.
F1 HF Tumor Histopathology
34%
66%
Benign
Malignant
F3 HF Tumor Histopathology
23%
77%
