# Supplementary figures and images for: Maternal intake of high n-6 polyunsaturated fatty acid diet during pregnancy causes transgenerational increase in mammary cancer risk in mice
Source: Breast Cancer Res. 2017 Jul 3;19:77. doi: 10.1186/s13058-017-0866-x (PMC5494892; doi:10.1186/s13058-017-0866-x)

**A.****WT F1 Tumor Multiplicity**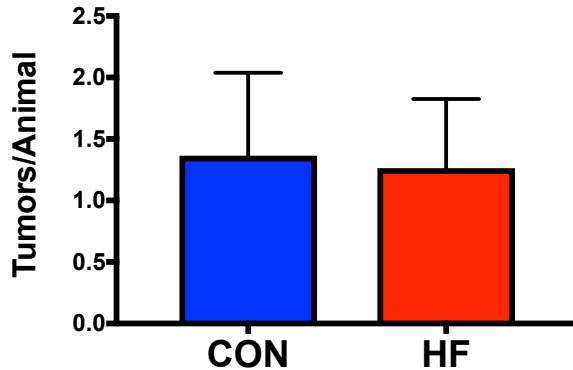**B.****WT F3 Tumor Multiplicity**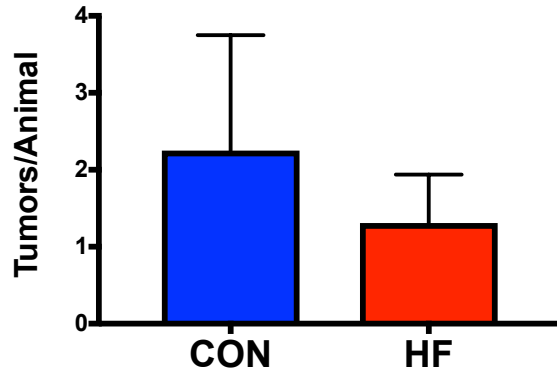

Supplement: Supplementary file 4 — Figure S2. Mammary tumor multiplicity was not altered between (a) F1 control (CON; n = 30 mice) and high-fat (HF; n = 29 mice) offspring or (b) F3 CON (n = 19 mice) and HF (n = 24 mice) generation offspring. (PDF 54 kb) [file 13058_2017_866_MOESM4_ESM.pdf]
