# Supplementary material for: Maternal intake of high n-6 polyunsaturated fatty acid diet during pregnancy causes transgenerational increase in mammary cancer risk in mice
Source: Breast Cancer Res. 2017 Jul 3;19:77. doi: 10.1186/s13058-017-0866-x (PMC5494892; doi:10.1186/s13058-017-0866-x)
Supplement: Supplementary file 5 — Table S3. Common differentially expressed genes in the mammary glands of F1 and F3 generation offspring of dams fed high-fat n-6 PUFA diet during pregnancy, compared with control mice. (DOCX 104 kb) [file 13058_2017_866_MOESM5_ESM.docx]

**Table S3.** Common differentially expressed genes in F1 and F3 generation offspring of dams fed high fat n-6 PUFA diet during pregnancy.

| **Gene ID** | **F1** | | | **F3** | | |
| --- | --- | --- | --- | --- | --- | --- |
|  | **Log fold change** | | **p-value** | **Log fold change** | | **p-value** |
| Akr1c14 | -0.690 |  | 0.006 | -1.020 |  | 0.006 |
| Akt2 | 0.457 |  | 0.017 | 0.231 |  | 0.042 |
| Alb | -1.010 |  | 0.010 | -0.788 |  | 0.014 |
| Alg6 | 0.450 |  | 0.023 | 0.376 |  | 0.042 |
| Amigo3 | 0.327 |  | 0.014 | 0.546 |  | 0.010 |
| Ankef1 | -0.318 |  | 0.033 | -0.348 |  | 0.009 |
| Atp5sl | 0.356 |  | 0.018 | 0.503 |  | 0.024 |
| Atp6v1g2 | 0.697 |  | 0.016 | 0.659 |  | 0.027 |
| Ccl8 | -0.756 |  | 0.044 | -1.317 |  | 0.004 |
| Cdh24 | 0.622 |  | 0.015 | 0.821 |  | 0.036 |
| Cdkn1a | -0.912 |  | 0.048 | -1.183 |  | 0.034 |
| Dpf3 | 0.935 |  | 0.005 | 0.825 |  | 0.009 |
| Egr3 | 0.963 |  | 0.002 | 0.953 |  | 0.023 |
| Ephb3 | 0.409 |  | 0.039 | 0.696 |  | 0.031 |
| Faap100 | 0.407 |  | 0.015 | 0.263 |  | 0.043 |
| Gdpd2 | -1.946 |  | 0.043 | -0.963 |  | 0.021 |
| Gpcpd1 | 0.377 |  | 0.045 | 0.254 |  | 0.038 |
| Grhl3 | 0.894 |  | 0.026 | 1.457 |  | 0.032 |
| Hes1 | 1.103 |  | 0.021 | 0.875 |  | 0.045 |
| Id4 | 0.497 |  | 0.032 | 1.362 |  | 0.003 |
| Ier5l | 0.940 |  | 0.008 | 0.779 |  | 0.012 |
| Igfbp6 | -0.829 |  | 0.007 | -0.578 |  | 0.022 |
| Itih4 | 1.017 |  | 0.026 | 1.089 |  | 0.045 |
| Izumo4 | 0.340 |  | 0.047 | 0.390 |  | 0.050 |
| Jam3 | 0.307 |  | 0.009 | 0.325 |  | 0.009 |
| Lfng | 0.374 |  | 0.014 | 0.389 |  | 0.046 |
| Lppr2 | 0.463 |  | 0.011 | 0.511 |  | 0.035 |
| Magix | 0.773 |  | 0.019 | 0.897 |  | 0.000 |
| mt-Ts2 | -2.078 |  | 0.016 | -0.664 |  | 0.037 |
| Oas3 | -0.766 |  | 0.018 | -0.884 |  | 0.004 |
| Optc | 0.662 |  | 0.042 | 0.601 |  | 0.018 |
| Parp8 | 0.269 |  | 0.034 | 0.453 |  | 0.008 |
| Pcdhga8 | 0.354 |  | 0.009 | 0.258 |  | 0.032 |
| Rnase10 | -0.743 |  | 0.013 | -1.246 |  | 0.006 |
| Sema5b | 0.791 |  | 0.022 | 0.853 |  | 0.025 |
| Slc26a10 | 1.114 |  | 0.005 | 1.060 |  | 0.003 |
| Slc26a3 | 0.966 |  | 0.030 | 2.172 |  | 0.031 |
| Slc5a3 | -0.739 |  | 0.003 | -0.578 |  | 0.025 |
| Slc6a2 | -1.048 |  | 0.010 | -1.115 |  | 0.012 |
| Slc6a9 | 0.406 |  | 0.025 | 0.523 |  | 0.027 |
| Slfn1 | -0.857 |  | 0.043 | -1.179 |  | 0.002 |
| Snora41 | 1.841 |  | 0.010 | 0.733 |  | 0.040 |
| St8sia2 | 0.960 |  | 0.020 | 2.104 |  | 0.002 |
| Tbx2 | 0.607 |  | 0.016 | 0.630 |  | 0.019 |
| Tcea2 | 0.397 |  | 0.018 | 0.837 |  | 0.002 |
| Zbp1 | -0.531 |  | 0.043 | -0.596 |  | 0.015 |
| Zfp467 | 0.435 |  | 0.034 | 0.392 |  | 0.035 |
| Zfp683 | -1.226 |  | 0.032 | -1.380 |  | 0.001 |
