# Supplementary material for: Maternal intake of high n-6 polyunsaturated fatty acid diet during pregnancy causes transgenerational increase in mammary cancer risk in mice
Source: Breast Cancer Res. 2017 Jul 3;19:77. doi: 10.1186/s13058-017-0866-x (PMC5494892; doi:10.1186/s13058-017-0866-x)
Supplement: Supplementary file 6 — Table S4. Top differentially expressed pathways and predicted upstream regulators in F1 and F3 offspring of control or high-fat fed dams, identified in Ingenuity Pathway Analysis. (DOCX 47 kb) [file 13058_2017_866_MOESM6_ESM.docx]

**Table S4.** Top differentially expressed pathways and predicted upstream regulators in F1 and F3 offspring of control or high fat fed dams, identified in Ingenuity Pathway Analysis.

| **Top Pathways** | **p-value** |
| --- | --- |
| VDR/RXR Activation | 7.03 x 10^-4^ |
| PTEN Signaling | 2.32 x 10^-3^ |
| FXR/RXR Activation | 2.79 x 10^-3^ |
| Hereditary Breast Cancer Signaling | 2.92 x 10^-3^ |
| Notch Signaling | 3.25 x 10^-3^ |
|  |  |
| **Top Predicted Upstream Regulators** | **p-value** |
| IRF7 | 2.11 x 10^-5^ |
| DLL3 | 2.61 x 10^-5^ |
| JAG1 | 4.34 x 10^-5^ |
| IRF3 | 6.61 x 10^-5^ |
| MSGN1 | 9.09 x 10^-5^ |
