# Supplementary material for: Maternal intake of high n-6 polyunsaturated fatty acid diet during pregnancy causes transgenerational increase in mammary cancer risk in mice
Source: Breast Cancer Res. 2017 Jul 3;19:77. doi: 10.1186/s13058-017-0866-x (PMC5494892; doi:10.1186/s13058-017-0866-x)
Supplement: Supplementary file 7 — Table S5. Top differentially expressed diseases and biofunctions between F1 and F3 offspring of dams fed control or high-fat diet during pregnancy, identified using Ingenuity Pathway Analysis. (DOCX 65 kb) [file 13058_2017_866_MOESM7_ESM.docx]

**Table S5**. Top differentially expressed diseases and biofunctions between F1 and F3 offspring of dams fed control or high fat diet during pregnancy, identified using Ingenuity Pathway Analysis.

| **Name** | **p-value** |
| --- | --- |
| Embryonic development | 2.43 x 10^-2^ – 3.24 x 10^-6^ |
| Lymphoid tissue structure and development | 1.87 x 10^-2^ – 3.24 x 10^-6^ |
| Organ development | 2.43 x 10^-2^ – 3.24 x 10^-6^ |
| Organismal development | 2.43 x 10^-2^ – 3.24 x 10^-6^ |
| Tissue development | 2.43 x 10^-2^ – 3.24 x 10^-6^ |
| Cellular growth and proliferation | 2.43 x 10^-2^ – 1.46 x 10^-5^ |
| Cellular development | 2.43 x 10^-2^ – 9.80 x 10^-5^ |
| Cellular function and Maintenance | 1.74 x 10^-2^ – 9.80 x 10^-5^ |
| Cell morphology | 1.99 x 10^-2^ – 1.74 x 10^-4^ |
| Cell cycle | 2.43 x 10^-2^ – 3.74 x 10^-4^ |
| Respiratory disease | 2.21 x 10^-2^ – 3.04 x 10^-4^ |
| Cancer | 2.21 x 10^-2^ – 6.19 x 10^-4^ |
| Organismal injury and abnormalities | 2.43 x 10^-2^ – 6.19 x 10^-4^ |
| Tumor morphology | 1.99 x 10^-2^ – 6.19 x 10^-4^ |
| Inflammatory Response | 2.43 x 10^-2^ – 1.71 x 10^-3^ |
