# Supplementary material for: Maternal intake of high n-6 polyunsaturated fatty acid diet during pregnancy causes transgenerational increase in mammary cancer risk in mice
Source: Breast Cancer Res. 2017 Jul 3;19:77. doi: 10.1186/s13058-017-0866-x (PMC5494892; doi:10.1186/s13058-017-0866-x)
Supplement: Supplementary file 8 — Supplementary reference list for KDDN. (DOCX 102 kb) [file 13058_2017_866_MOESM8_ESM.docx]

**Supplementary Reference List for KDDN**

1. Eklund EA, Sun L, Yang SP, Pasion RM, Thorland EC, Freeze HH. Congenital disorder of glycosylation Ic due to a de novo deletion and an hALG-6 mutation. Bioch Bioph Res Co. 2006;339:755–60.
2. Hoyal CR, Kammerer S, Roth RB, Reneland R, Marnellos G, Kiechle M, et al. Genetic polymorphisms in DPF3 associated with risk of breast cancer and lymph node metastases. J Carcinog. 2005;4:13–9.
3. Theodorou M, Speletas M, Mamara A, Papachristopoulou G, Lazou V, Scorilas A, et al. Identification of a STAT5 target gene, Dpf3, provides novel insights in chronic lymphocytic leukemia. PLoS ONE. 2013;8:e76155–10.
4. Zhao P, Guo S, Tu Z, Di L, Zha X, Zhou H, et al. Grhl3 induces human epithelial tumor cell migration and invasion via downregulation of E-cadherin. Acta Biochim Biophys Sin. 2016;48:266–74.
5. Xu H, Liu C, Zhao Z, Gao N, Chen G, Wang Y, et al. Clinical implications of GRHL3 protein expression in breast cancer. Tumor Biol. 2013;35:1827–31.
6. Le Goff MM, Sutton MJ, Slevin M, Latif A, Humphries MJ, Bishop PN. Opticin exerts its anti-angiogenic activity by regulating extracellular matrix adhesiveness. J Biol Chem. 2012;287:28027–36.
7. Mikaelsson E, Osterborg A, Fard ZT, Mahmoudi A, Mahmoudian J, Jeddi-Tehrani M, et al. Opticin, a small leucine-rich proteoglycan, is uniquely expressed and translocated to the nucleus of chronic lymphocytic leukemia cells. Exp Hematol. 2013;2:23.
8. Bellemare V, Labrie F, Luu-The V. Isolation and characterization of a cDNA encoding mouse 3α-hydroxysteroid dehydrogenase. J Steroid Biochem. 2006;98:18–24.
9. Tuppen HA, Naess K, Kennaway NG, Al-Dosary M, Lesko N, Yarham JW, et al. Mutations in the mitochondrial tRNASer(AGY) gene are associated with deafness, retinal degeneration, myopathy and epilepsy. Eur J Hum Genet. 2012;20:897–904.
10. Lin X, Ma L, Fitzgerald RL, Ostlund RE Jr. Human sodium/inositol cotransporter 2 (SMIT2) transports inositols but not glucose in L6 cells. Arch Biochem Biophys. 2009;481:197–201.
11. Torres GE, Gainetdinov RR, Caron MG. Plasma membrane monoamine transporters: structure, regulation and function. Nat Rev Neurosci. 2003;4:13–25.
12. Li B, Wan X, Zhu Q, Li L, Zeng Y, Hu D, et al. Net expression inhibits the growth of pancreatic ductal adenocarcinoma cell PL45 in vitro and in vivo. PLoS ONE. 2013;8:e57818–9.
13. Sloan KA, Marquez HA, Li J, Cao Y, Hinds A, O'Hara CJ, et al. Increased PEA3/E1AF and decreased Net/Elk-3, both ETS proteins, characterize human NSCLC progression and regulate caveolin-1 transcription in Calu-1 and NCI-H23 NSCLC cell lines. Carcinogenesis. 2009;30:1433–42.
14. Takaoka A, Wang Z, Choi MK, Yanai H, Negishi H, Ban T, et al. DAI (DLM-1/ZBP1) is a cytosolic DNA sensor and an activator of innate immune response. Nature. 2007;448:501–5.
15. Wang Z, Choi MK, Ban T, Yanai H, Negishi H, Lu Y, et al. Regulation of innate immune responses by DAI (DLM-1/ZBP1) and other DNA-sensing molecules. PNAS. 2008;105:5477–82.
16. Lladser A, Mougiakakos D, Tufvesson H, Ligtenberg MA, Quest AF, Kiessling R, et al. DAI (DLM-1/ZBP1) as a genetic adjuvant for DNA vaccines that promotes effective antitumor CTL immunity. Mol Ther. 2009;19:594–601.
17. Hirota E, Yan L, Tsunoda T, Ashida S, Fujime M, Shuin T, et al. Genome-wide gene expression profiles of clear cell renal cell carcinoma: Identification of molecular targets for treatment of renal cell carcinoma. Int J Oncol. 2006;29:799–827.
18. He L, Kernogitski Y, Kulminskaya I, Loika Y, Arbeev KG, Loiko E, et al. Pleiotropic meta-analyses of longitudinal studies discover novel genetic variants associated with age-related diseases. Front Genet. 2016;7:33–16.
19. Jin HJ, Jung S, DebRoy AR, Davuluri RV. Identification and validation of regulatory SNPs that modulate transcription factor chromatin binding and gene expression in prostate cancer. Oncotarget. 2016;7:54616-26.
20. Fleischmann KK, Pagel P, Schmid I, Roscher AA. RNAi-mediated silencing of MLL-AF9 reveals leukemia-associated downstream targets and processes. Mol Cancer. 2014;13:1–14.
21. Amé J-C, Spenlehauer C, de Murcia G. The PARP superfamily. Bioessays. 2004;26:882–93.
22. Molyneux G, Geyer FC, Magnay F-A, McCarthy A, Kendrick H, Natrajan R, et al. BRCA1 basal-like breast cancers originate from luminal epithelial progenitors and not from basal stem cells. Cell Stem Cell. 2010;7:403–17.
23. Baker LA, Holliday H, Swarbrick A. ID4 controls luminal lineage commitment in normal mammary epithelium and inhibits BRCA1function in basal-like breast cancer. Endocr Relat Cancer. 2016;23:R381–92.
24. Huang THM, Esteller M. Chromatin remodeling in mammary gland differentiation and breast tumorigenesis. Perspect Biol. 2010;2:a004515–5.
25. Lesjak MS, Marchan R, Stewart JD, Rempel E, Rahnenführer J, Hengstler JG. EDI3 links choline metabolism to integrin expression, cell adhesion and spreading. Cell Adhes Migr. 2014;8:499–508.
26. Kaulsay KK, Ng EH, Ji CY, Ho GH, Aw TC, Lee KO. Serum IGF-binding protein-6 and prostate specific antigen in breast cancer. Eur J Endocrin. 1999;140:164–8.
27. Bach LA, Fu P, Yang Z. Insulin-like growth factor-binding protein-6 and cancer. Clin. Sci. 2013;124:215–29.
28. Kiss AM, Jady BE, Bertrand E, Kiss T. Human box H/ACA pseudouridylation guide RNA machinery. Mol Cell Biol. 2004;24:5797–807.
29. Bao L, Zhang Y, Wang J, Wang H, Dong N, Su X, et al. Variations of chromosome 2 gene expressions among patients with lung cancer or non-cancer. Cell Biol Toxicol. 2016;32:419–35.
30. de Ronde JJ, Lips EH, Mulder L, Vincent AD, Wesseling J, Nieuwland M, et al. SERPINA6, BEX1, AGTR1, SLC26A3, and LAPTM4B are markers of resistance to neoadjuvant chemotherapy in HER2-negative breast cancer. Breast Cancer Res Tr. 2012;137:213–23.
31. Suzuki T, Inoue A, Miki Y, Moriya T, Akahira JI, Ishida T, et al. Early growth responsive gene 3 in human breast carcinoma: a regulator of estrogen-meditated invasion and a potent prognostic factor. Endocr Relat Cancer. 2007;14:279–92.
32. Vareslija D, McBryan J, Fagan A, Redmond AM, Hao Y, Sims AH, et al. Adaptation to AI therapy in breast cancer can induce dynamic alterations in ER activity resulting in estrogen-independent metastatic tumors. Clin Cancer Res. 2016;22:2765–77.
33. To SQ, Knower KC, Clyne CD. NFkB and MAPK signalling pathways mediate TNFa-induced Early Growth Response gene transcription leading to aromatase expression. Biochem Biophysl Res Co. 2013;433:96–101.
34. Sumitomo S, Fujio K, Okamura T, Morita K, Ishigaki K, Suzukawa K, et al. Transcription factor Early Growth Response 3 is associated with the TGF-1 expression and the regulatory activity of CD4-positive T cells in vivo. J Immunol. 2013;191:2351–9.
35. Huang Y, Li Z, Zhong Q, Li G, Zhang Y, Huang Z. Association of TBX2 and P21 expression with clinicopathological features and survival of laryngeal squamous cell carcinoma. Int J Clin Exp Med. 2014;7:5394–02.
36. Yu H, Liu B, Liu A, Li K, Zhao H. T-box 2 expression predicts poor prognosis in gastric cancer. Oncol Lett. 2015;:1–5.
37. Douglas NC, Papaioannou VE. The T-box transcription factors TBX2 and TBX3 in mammary gland development and breast cancer. J Mammary Gland Biol. 2013;18:143–7.
38. Wansleben S, Davis E, Peres J, Prince S. A novel role for the anti-senescence factor TBX2 in DNA repair and cisplatin resistance. Cell Death Disease. 2013;4:e846–9.
39. Vieira Braga FA, Hertoghs KML, Kragten NAM, Doody GM, Barnes NA, Remmerswaal EBM, et al. Blimp-1 homolog Hobit identifies effector-type lymphocytes in humans. Eur J Immunol. 2015;45:2945–58.
40. An CH, Je EM, Yoo NJ, Lee SH. Frameshift mutations of cadherin genes DCHS2, CDH10 and CDH24 genes in gastric and colorectal cancers with high microsatellite instability. Pathol Oncol Res. 2014;21:181–5.
41. Kirov G, Georgieva L, Williams N, Nikolov I, Norton N, Toncheva D, et al. Variation in the protocadherin γ A gene cluster. Genomics. 2003;82:433–40.
42. Theofilopoulos S, Lykidis A, Leondaritis G, Mangoura D. Novel function of the human presqualene diphosphate phosphatase as a type II phosphatidate phosphatase in phosphatidylcholine and triacylglyceride biosynthesis pathways. Biochim Biophys Acta. 2008;doi:10.1016/j.bbalip.2008.09.001.
43. Quach JM, Walker EC, Allan E, Solano M, Yokoyama A, Kato S, et al. Zinc finger protein 467 is a novel regulator of osteoblast and adipocyte commitment. J Biol Chem. 2011;286:4186–98.
44. Huang Y, Leung JWC, Lowery M, Matsushita N, Wang Y, Shen X, et al. Modularized functions of the fanconi anemia core complex. Cell Reports. 2014;7:1849–57.
45. Ferguson JF, Xue C, Hu Y, Li M, Reilly MP. Adipose tissue RNASeq reveals novel gene–nutrient interactions following n-3 PUFA supplementation and evoked inflammation in humans. J Nutr Biochem. 2016;30:126–32.
46. Hao S, Yang Y, Liu Y, Yang S, Wang G, Xiao J, et al. JAM-C promotes lymphangiogenesis and nodal metastasis in non-small cell lung cancer. Tumor Biol. 2014;35:5675–87.
47. Hajjari M, Behmanesh M, Sadeghizadeh M, Zeinoddini M. Junctional adhesion molecules 2 and 3 may potentially be involved in progression of gastric adenocarcinoma tumors. Med Oncol. 2013;30:380–7.
48. Leinster DA, Colom B, Whiteford JR, Ennis DP, Lockley M, McNeish IA, et al. Endothelial cell junctional adhesion molecule C plays a key role in the development of tumors in a murine model of ovarian cancer. FASEB J. 2013;27:4244–53.
49. Liu X, Mitrovic AD, Vandenberg RJ. Glycine transporter 1 associates with cholesterol-rich membrane raft microdomains. Biochem Biophys Res Co. 2009;384:530–4.
50. Bhutia YD, Babu E, Ramachandran S, Yang S, Thangaraju M, Ganapathy V. SLC transporters as a novel class of tumour suppressors: identity, function and molecular mechanisms. Biochem J. 2016;473:1113–24.
